# Rapid and non-destructive classification of salinity levels in brined kimchi cabbage using hyperspectral imaging

**DOI:** 10.1016/j.heliyon.2024.e40817

**Published:** 2024-11-29

**Authors:** Hyeyeon Song, Myounghwan Kim, Kwang Sun Yoo, Ji-Hyoung Ha

**Affiliations:** a Hygienic Safety and Materials Research Group, World Institute of Kimchi, 86 Kimchi-ro, Nam-gu, Gwangju, Republic of Korea; b AI Research, Elroilab Co., Ltd., 28 Digital-ro 30-gil, Guro-gu, Seoul, Republic of Korea

**Keywords:** Brined kimchi cabbage, Classification, Hyperspectral imaging, Kimchi

## Abstract

In this study, we demonstrate the potential of a non-destructive hyperspectral imaging processing method in the near-infrared (NIR) region (874–1734 nm) for classifying the quality of brined kimchi cabbage. The salinity level of brined kimchi cabbage is an important indicator of consumer preference and the quality of kimchi. Hence, we compared the water content and salinity of brined kimchi cabbage via hyperspectral data. We extracted the optimal wavelengths from the hyperspectral image dataset to classify the salinity level of the predicted brined kimchi cabbage, and thus, established a novel approach for classifying kimchi samples into quality-unacceptable and quality-acceptable groups. Standard normal variate and multiplicative scatter correction (MSC) were used for pathlength correction. The Savitzky–Golay first and second derivatives were used for the deconvolution of the raw spectral data. The experimental results confirmed that the decision tree model combined with MSC pathlength correction and Savitzky–Golay first derivative preprocessing was the best classification model. The proposed hyperspectral image–NIR system can be applied to the detection of salinity during industrial kimchi manufacturing.

## Highlights

• Hyperspectral imaging in near-infrared region (874–1734 nm).
• Classification into quality-acceptable and -unacceptable groups.
• Salinity of 1.0–1.8 % classified as proper-brined kimchi cabbage.
• Decision tree: multiplicative scatter correction, Savitzky–Golay 1st derivative.
• Precision, recall, and accuracy as evaluation indicators for classification model.

## 1 Introduction

Kimchi, an important product in the Korean diet, is a complex fermented food prepared by adding vegetables, such as cabbage and radish, to salted seafood, seasoning, and spices. It is known as a functional food with nutritional properties as well as anticancer, antioxidant, and immune-boosting effects [1]. Kimchi is produced via the following steps: brining, soaking, maturation, and storage. Brining, which is the first step in kimchi production, has a decisive influence on the quality characteristics of kimchi such as taste, smell, and texture [2]. The temperature of the brining water, brining time, and salinity of the brining water affect the brining process. During the brining process, salt induces osmosis between the cabbage tissues, suppressing the growth of harmful microorganisms and facilitating the growth of salt-tolerant microorganisms as the dominant bacteria, as they are essential for kimchi fermentation [3]. In addition, substances such as pectin, the main component of cabbage tissue, decompose, whereas water-soluble vitamins, sugars, sulfur-containing substances, and free amino acids are eluted, which also affects the taste of kimchi [4].

Brined kimchi cabbage is an indispensable ingredient of kimchi, an authentic Korean fermented food established by the Codex Alimentarius Commission [31]. Brined kimchi cabbage accounts for more than 70 % of kimchi ingredients; therefore, brined kimchi cabbage directly influences kimchi quality [5]. The salinity of brined kimchi cabbage must be classified into a specific range to standardize kimchi quality. A traditional method for measuring the salinity of brined kimchi cabbage is the Mohr method, employing simple devices such as an electrical conductivity sensor and a salinity meter. However, the quantity of brined kimchi cabbage produced by the brining process is so large that individually measuring the salinity of all samples is difficult. Nevertheless, the number of brined kimchi cabbage samples can be reduced through a systematic sampling method; however, this specific sampling technique must consider sample bias. Furthermore, such methods are destructive and do not meet current detection requirements. Therefore, the development of a rapid and non-destructive detection method based on image analysis technology to maintain kimchi quality is urgently required.

Hyperspectral imaging is an innovative platform that can provide spatial and spectral information in real time by integrating computer vision [6] and spectroscopy [7]. Hyperspectral imaging techniques can provide useful information regarding the interactions between the food matrix and electromagnetic radiation. This information allows a non-invasive analysis of the chemical properties of corresponding materials. Hence, researchers have used hyperspectral image extensively for food quality management, classification, and safety in recent decades [8,9]. Hyperspectral imaging technology has also been employed to evaluate firmness, bruising, and defects in food [10,11], to quantify various food chemical components [12], to assess freshness, and to monitor the quality [10,11] and soluble solids content [13,14]. Although various hyperspectral imaging laboratory-scale experiments have been performed to classify the spectral characteristics of fruits, vegetables, frozen foods, and meat products [[15], [16], [17]], no studies have focused on classifying the salinity of brined kimchi cabbage.

Hyperspectral imaging techniques exhibit enormous potential as food quality control tools owing to the diverse chemometric and mathematical methods available for use with the spectral information that they provide. Although hyperspectral imaging techniques are effective, there are obstacles to their application in real-time monitoring applications, primarily due to the complexity of processing large and massive hyperspectral image datasets. There are various effectively used approaches adopted for analyzing massive hyperspectral image data, including the multilayer perceptron (MLP), random forest (RF), eXtreme Gradient Boosting (XGBoost), k-nearest neighbors (K-NN), naive Bayes classifier (NBC), and decision tree (DT). In this study, we explored the feasibility of non-destructive HSI in the near-infrared (NIR) region to classify the salinity of brined kimchi cabbage. The specific objectives were to compare the moisture content of the brined kimchi cabbage samples based on the data obtained from hyperspectral image, to screen the hyperspectral image characteristics of the samples in the 900 to 1700 nm spectral range to distinguish their salinity, and to introduce a novel approach for the classification of properly brined kimchi cabbage and under-/over-brined kimchi cabbage. This study aimed to show that hyperspectral image is a non-destructive, fast, simple, and robust technique for predicting the salinity of brined kimchi cabbage.

## 2 Materials and methods

### 2.1 Preparation of brined kimchi cabbage samples

**Fig. 1:**
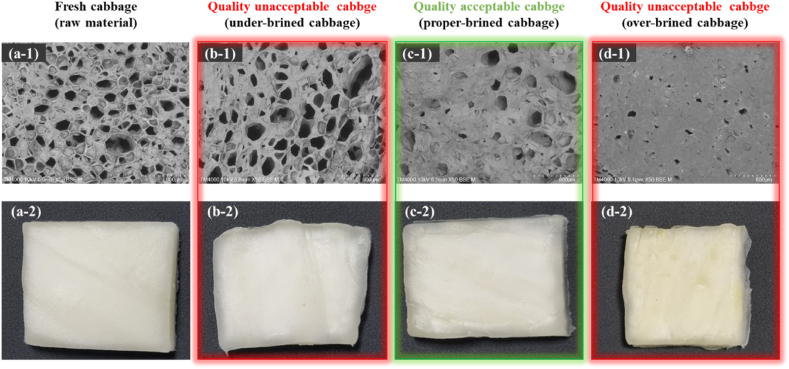
Scanning electron microscopy images of cabbage samples and cut brined kimchi cabbage at different brining stages: (a-1 and a-2) fresh cabbage, (b-1 and b-2) UBC, (c-1 and c-2) PBC, and (d-1 and d-2) OBC.

Kimchi cabbages were purchased from an agricultural wholesale market (Gwangju, South Korea). Refined salt (hanjusalt, Korea) was used to prepare the brined kimchi cabbage. Fresh, intact kimchi cabbages were cut into 3 × 3 cm^2^ slices (approximately 0.3 mm thickness and 7 g) with a sterile stainless-steel knife and were used immediately at 20 °C. The brined kimchi cabbage was prepared under specific conditions involving the brining time (14 h), brine concentration (14 %), and brine temperature (20 °C). The sliced brined kimchi cabbage was salted in brine, washed three times with fresh water, and then drained for 3 h. Subsequently, the kimchi cabbage was placed in a customized water bath (1650 mm (W) × 800 mm (L) × 750 mm (D); AsungTECH, Seoul, Korea) at a constant temperature. The 180 brined kimchi cabbage samples were classified into two groups of 90 samples: a quality-acceptable group (QAG) and a quality-unacceptable group (QUG) based on the following three salinity standards: brined kimchi cabbage with a salinity of less than 1 % (under-brined kimchi cabbage, UBC), brined kimchi cabbage with a salinity of more than 1 % and less than 1.8 % (proper-brined kimchi cabbage, PBC), and brined kimchi cabbage with a salinity of more than 1.8 % (over-brined kimchi cabbage, OBC). The UBC and OBC groups were classified as the QUG, whereas the PBC group was classified as the QAG (Fig. 1). Fig. 1 shows the real sample images of fresh cabbage (a-1 and a-2), UBC (b-1 and b-2), PBC (c-1 and c-2), and OBC (d-1 and d-2). The salinity and water content of the brined kimchi cabbage in the three sample groups (UBC, PBC, and OBC) were within a reasonable range, and such wide variability of reference measurement data enabled the calibration model to be more stable and robust. A total of 135 brined kimchi cabbage samples were used for the calibration set and 45 samples were used as the prediction set (Table S1). Notably, the ranges of both the salinity and water content in the calibration set covered the range in the prediction set. Moreover, no significant difference was observed in the standard deviation values between the calibration and prediction sets, which was reasonable for further analysis and modeling.

### 2.2 Analysis of the physicochemical properties of the brined kimchi cabbage samples

#### 2.2.1 Determination of water content

Approximately 2–2.5 g of crushed kimchi cabbage samples was measured using an electronic scale and lyophilized in a freeze-dryer until a constant weight was observed. The water content of the samples was calculated using Equation (1).

(1) $$W=\frac{\left(W_{1}-W_{2}\right)}{W_{1}}\times 100\%\text{,}$$

where W is the water content of the brined kimchi cabbage sample, and W_1_ and W_2_ are the weights of the original and dry samples, respectively. For water content, 30 samples were used for each of the four groups.

#### 2.2.2 Determination of salinity

The salinity of the brined kimchi cabbage was measured using the Mohr method [30]. For salinity, 30 samples were used for each of the four groups. Briefly, 1000 mg of the homogenized brined kimchi cabbages was serially diluted 100-fold with distilled water followed by filtration. The filtered solution (10 mL) was mixed with 1 mL of 2 % K_2_CrO_4_ (Daejung, Seoul, Korea) and adjusted with 0.02 N AgNO_3_ (Daejung). The salinity of the brined kimchi cabbage was calculated using Equation (2).

(2) $$\text{Salinity}\;\left(\%\right)=\frac{{0.02\;N\;\text{AgNO}}_{3}\;\left(\text{mL}\right)\times 0.00117\times {\text{AgNO}}_{3}\;\text{factor}\times \text{dilution}\;\text{rate}}{\text{Brined}\;\text{kimchi}\;\text{cabbage}\;s\text{ample}\;\left(g\right)}\times 100\text{.}$$

### 2.3 Hyperspectral image data cube acquisition and correction

#### 2.3.1 Hyperspectral image system

A line scanning-type (push-broom scanner) NIR hyperspectral imaging system (N17E-QE, SPECIM, Spectral Imaging Ltd., Oulu, Finland) was used to collect the hyperspectral image data cube of the brined kimchi cabbages. The NIR–hyperspectral image system consisted of a short-wave infrared (SWIR) camera with an OLES56 camera lens (SWIR-CL-400-N25E, SPECIM), covering a spectral wavelength range of 874–1734 nm with 320 × 256 pixels. A translation scanner with a wavelength of approximately 12 nm was used. The system consisted of a transfer plate driven by a stepper motor (Isuzu Optics Corp., Taiwan) and two symmetrically fixed 150 W tungsten halogen lamps (Fiber-Lite DC950 Illuminator, Dolan Jenner Industries Inc., Boxborough, MA, USA). Both sides of the camera were used as illumination sources at an angle of 45°. The NIR–hyperspectral imaging system was placed in a dark room and controlled using a computer. For each sample scan, each spectral image was acquired for 3 min at a controlled ambient temperature (15 °C) in a dark chamber.

#### 2.3.2 Hyperspectral image data acquisition

To obtain clear and non-deformable images containing whole brined kimchi cabbages, the following three parameters that significantly affect hyperspectral image data must be set accurately: the scanning speed (21.7 mm/s) of the conveying plate, the exposure time of the hyperspectral sensor, and the distance (29 cm) between the sensor and brined kimchi cabbage sample. Hence, these parameters were controlled using an ENVI® modeler. For image calibration, hyperspectral and dark/white reference images were acquired under identical experimental conditions. Finally, corrected images were calculated using Equation (3).

(3) $$I_{C}=\left[\;\frac{I_{O}-I_{D}}{I_{W}-I_{D}}\;\right]\times 100$$

where *I*_*C*_ is the calibrated reflectance value, *I*_*D*_ is the dark reference image, *I*_*W*_ is the white reference image, and *I*_*O*_ is the original hyperspectral image. Sample spectral images were extracted using ENVI® 4.7 software (L3Harris Technologies, Boulder, CO, USA). For hyperspectral images acquired in the range of 938–1710 nm, the region of interest (ROI) must be predefined to extract the spectral information. The ROI of each sample was determined as an orthogonal section region of each kimchi cabbage, and the spectral data of the ROI were extracted. Finally, the mean spectral information of the individual samples was acquired by averaging all pixels of the ROI for additional analysis.

### 2.4 Model development and performance evaluation

#### 2.4.1 Data preprocessing and data analysis

Data preprocessing was performed to improve the accuracy of the classification model and the impact of irregularities in the spectral data owing to the sample texture, light scattering, and random noise. The hyperspectral image data were preprocessed using BigZami software (CSLEE®, Seoul, Korea). The standard normal variate (SNV) method and multiplicative scatter correction (MSC) were used to compensate for differences in the sample pathlength (pathlength correction). The Savitzky–Golay derivative method, including the first and second derivatives, was used to reveal peaks that appear as shoulders and to determine the exact center of the shoulders in the raw spectra to ensure model reliability.

#### 2.4.2 Model development and selection of optimal wavelengths

Although hyperspectral images can provide a wealth of information, the analysis of spectral data is challenging due to their collinearity and redundancy. In particular, the classification model may exhibit convergence instability because high-dimensional imaging data are affected by a high degree of interband correlation, which induces data redundancy. To overcome these technical limitations, we selected the optimal wavelength from the hyperspectral image data cubeto minimize the data processing complexity, simplify the model, and develop a robust, reliable classification model. We used algorithms for solving integer programs to select the optimal wavelength band that is suitable for use in multispectral imaging systems, which is the most common method. Considering the variations among the different optimal selection methods, the MLP, RF, XGBoost, K-NN, NBC, and DT were compared to select the optimal wavelengths.

### 2.5 Multivariate data analysis and model performance evaluation

The model performance was evaluated using five metrics: accuracy, specificity, F1 score, precision, and sensitivity. The accuracy indicates the ratio of the sum of true positives and true negatives to the sum of all positives and negatives. The specificity is the ratio of true negatives to all actual negatives. The precision is the ratio of true positives to all elements identified as positive (including false positives). The recall reflects the ratio of true positives to all relevant elements (i.e., actual positives). The F1 score indicates the harmonic mean of the recall and precision and expresses the classification accuracy in unbalanced datasets. The five metrics were calculated using Equations (4), (5), (6), (7), (8).

(4) $$Accuracy=\frac{\left(True\;positives+True\;negatives\right)}{\left(True\;positives+True\;negatives+False\;positives+False\;negatives\right)}$$

(5) $$Specificity=\frac{True\;negatives}{\left(True\;negarives+False\;positives\right)}$$

(6) $$Precision=\frac{True\;positives}{\left(True\;positives+False\;positives\right)}$$

(7) $$Recall=\frac{True\;positives}{\left(True\;positives+False\;negatives\right)}$$

(8) $$F1\;score=\frac{\left(2\times Precision\times Recall\right)}{\left(Precision+Recall\right)}$$

### 2.6 Visualization maps

The spectral data and spatial information based on each pixel in the hyperspectral image enabled the prediction of the chemical composition of each pixel by applying our powerful classification model. The model with the least variable number and most optimal performance was used to form distribution maps of the water content concentrations in the entire piece of brined kimchi cabbage. These hyperspectral imaging visualization maps of the brined kimchi cabbage were obtained through hyperspectral image analysis and processing using ENVI®.

### 2.7 Field emission scanning electron microscopy (FE-SEM)

FE-SEM (SU-70; Hitachi, Tokyo, Japan) was used to visualize cut brined kimchi cabbage at different brining stages. According to processes explained previously [18], each FE-SEM sample was processed with some modifications. Each brined kimchi cabbage sample was washed three times with PBS, and cabbage tissue were fixed with 2.5 % glutaraldehyde (Sigma-Aldrich, St. Louis, MO, USA) in PBS. The fixed bacterial cells were continuously treated for 20 min each with ethanol concentrations of 50 %, 60 %, 70 %, 80 %, and 90 % once, then three times in 100 % concentration. Then concentrations of 33 %, 50 %, 66 %, and 100 % hexamethyldisilazane (Sigma-Aldrich) in ethanol were treated for sequential dehydration for 15 min each. The dehydrated FE-SEM samples were coated with platinum and observed by FE-SEM.

## 3 Results

### 3.1 Physicochemical properties of the brined kimchi cabbage samples

**Fig. 2:**
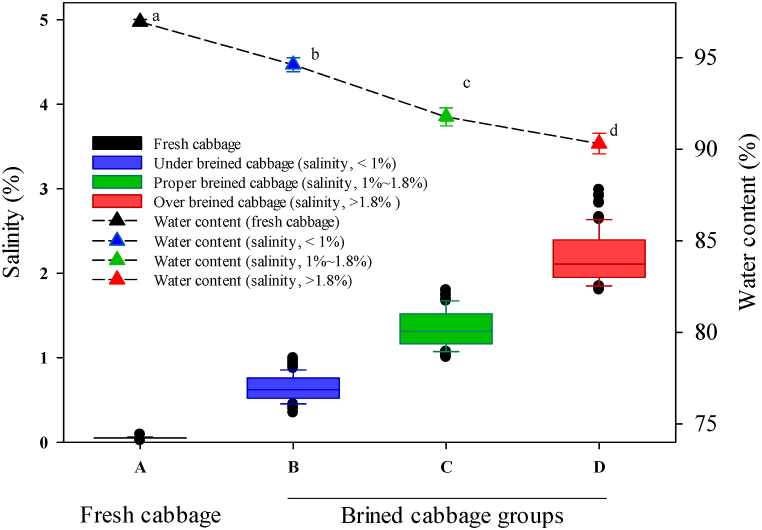
Water content and salinity for cabbage samples at different brining stages. (A) Fresh cabbage, (B) brined kimchi cabbage with a salinity of less than 1 % (under-brined kimchi cabbage, UBC), (C) brined kimchi cabbage with a salinity of more than 1 % and less than 1.8 % (proper-brined kimchi cabbage, PBC), and (D) brined kimchi cabbage with a salinity of more than 1.8 % (over-brined kimchi cabbage, OBC).

Following brining, the physicochemical properties of the brined kimchi cabbage were analyzed and the groups were classified by salinity level based on the experimental results. The descriptive statistics of the salinity and water content in the brined kimchi cabbage samples obtained from the three sample groups (UBC, PBC, and OBC) are shown in Fig. 2. Fresh cabbage samples exhibited the highest percentage of water content (96.96 ± 0.13) with significant differences, whereas the water contents of the UBC, PBC, and OBC groups were 94.62 ± 0.38, 91.77 ± 0.49, and 90.13 ± 0.38, respectively. The salinity values of the UBC, PBC, and OBC groups were 0.68 ± 0.19, 1.41 ± 0.25, and 2.29 ± 0.38, respectively. The significant difference in the water content of the brined kimchi cabbage could be attributed to osmotic action; overall, higher salinity results in a lower moisture content in brined kimchi cabbage. During the brining process, because of osmosis between the cabbage tissue and brine, pectin, which the main component of the cabbage epidermis, is decomposed; the cell membrane is destroyed; and water-soluble substances, such as vitamin C, sugars, sulfur compounds, and free amino acids, are eluted, filling the space with solid salt [19,20]. The cell membrane acts as a semi-permeable membrane, allowing moisture to move from the inside of the cell, where the salt concentration is low, to the outside, where the salt concentration is high. Among the quality standards of brined kimchi cabbage, salinity is a major factor that directly affects microbial growth. During the fermentation of kimchi, which is a non-heat-sterilized food, the growth of quality-degrading microorganisms cannot be controlled in brined kimchi cabbage with very low salinity, resulting in the growth of the pathogenic microorganism *Pectobacterium carotovorum* subsp. *carotovorum* (the causative agent of cabbage soft rot) [21]. In addition, brined kimchi cabbage with excessively high salinity negatively impacts consumer preference and causes consumer dissatisfaction owing to its irritating salty taste. Therefore, brined kimchi cabbage with a salinity of 1 %–1.8 %, that is, brined kimchi cabbage with an appropriate salinity, is essential to prevent these side effects.

### 3.2 Analysis of spectral characteristics

**Fig. 3:**
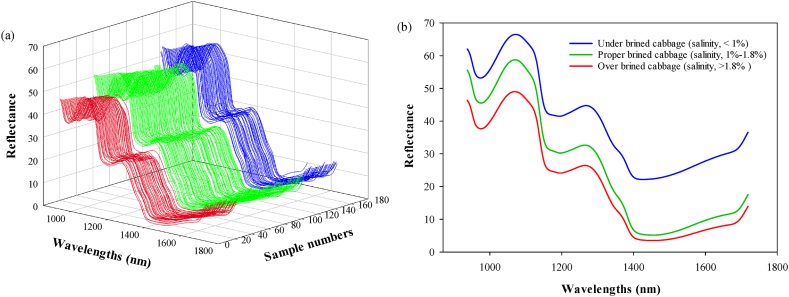
(a) Reflectance spectra of all brined kimchi cabbage samples and (b) average spectra of the UBC, PBC, and OBC samples.

The plots of the original hyperspectral image reflectance spectra of the brined kimchi cabbages at each salinity level are shown in Fig. 3. In the SWIR bands observed between 938 and 1711 nm, each spectrum contained 197 variables. Although the spectral curves of brined kimchi cabbage samples with different salinities were very similar, some differences existed in the spectral intensity of the reflectance at different wavelengths, indicating that the different sample groups had similar internal components (Fig. 3). Fig. 3(a) and (b) represents the reflectance spectra of UBC, PBC, and OBC of all samples and average spectra for three sample groups (UBC, PBC, and OBC), respectively. Similar spectral curves demonstrate that the phytochemical information obtained from the UBC, PBC, and OBC groups was similar, despite the complexity and diversity of the extrinsic/intrinsic factors that affected the salinity of the brined kimchi cabbage samples. These spectral data also indicate that a practical preprocessing step is required to extract the feature wavelengths that are useful for distinguishing them. Overall, brined kimchi cabbage samples with higher salinity had lower reflectance owing to their low water content. Salinity stress generally induces physiological responses in plants and can cause changes in metabolic activity [22]. The reflectance intensity of the reflectance spectrum of fresh produce was higher in the full-wave region than that of salted produce. Salinity stress could generally cause physiological responses in plants and induce changes in metabolic activity. Therefore, salinity stress affects the water distribution of kimchi cabbage, which could be a kind of adaptation mechanism to salt exposure. In addition, it is known that high-salt salting involves the release of water content from vegetable tissue cells by osmosis, and it is thought that this mechanism leads to differences in absorbance in specific wavelength regions.

Hyperspectral image techniques could be acceptable for non-destructive assessment of brining processes in real time because it provides both spectral and spatial dataset of sample by combining spectroscopic tools and hyperspectral image. However, application of any of hyperspectral image techniques has not been studied to date to monitor brined agricultural products including brined kimchi cabbages, to predict the brining salt concentration based on the correlation between physicochemical properties and hyperspectral data cube. Recently, hyperspectral image techniques have been mainly applied to monitor salinity during the brining process of sea cucumber, salmon fillet, and ham slice [23]. Sun et al. [22] analyzed the correlation between physicochemical properties of brined agricultural products and hyperspectral image data and reported significant absorption peaks in the region.

The absorption peaks at 976–980, 1069, 1180–1200, 1235–1240, and 1430–1450 nm (Fig. 3(b)) are related to the specific internal compounds of brined kimchi cabbage, that is, the water content in the cabbage [22]. The absorption peaks and valleys in the SWIR spectral data corresponded to the combination bands of O–H, C–H, and C–H2 in carbohydrates and water [24]. According to Wu & Sun [12] and Sun et al. [22], the absorption peaks at 976–980 and 1430–1450 nm correspond to the combination bands of O–H in the water molecules in vegetables. The absorption peaks at 1180–1200 and 1235–1240 nm are also associated with water sensitivity and moisture in agricultural crops [25,26]. Optimum waveband assignment plays a key role in identifying chemical components because hyperspectral image technology mainly relies on chemometric analysis to evaluate food quality attributes. The correlation between wavebands and molecular combination bands is key to understanding the chemistry behind the classification models [27]. Table S2 presents the specific waveband assignments for the water content and moisture assessment of various agricultural crops using hyperspectral image. The difference in the SWIR spectrum reflectance can potentially help to differentiate the physiochemical characteristics of brined kimchi cabbage samples with different degrees of water content and salinity. However, reflection peaks and several absorptions were detected in the wavebands, which were typically located in the spectral region with a wide frequency band, rendering direct extraction of the key variables of water content and salinity impossible. Therefore, an effective process is required to extract the feature wavelengths based on the optimum algorithm.

### 3.3 Establishing prediction models based on full wavelengths

**Fig. 4:**
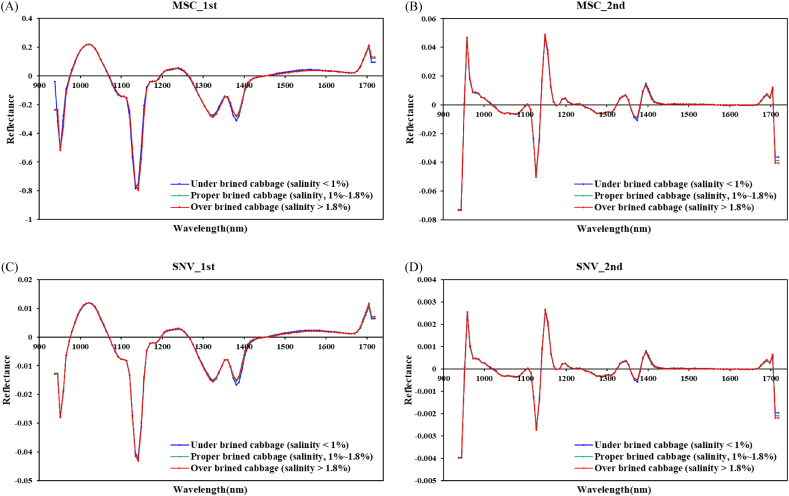
Spectral data handled by using different preprocessing methods: (A) first derivatives + multiple scattering corrections (MSC); (B) second derivatives + multiple scattering corrections (MSC); (C) first derivatives + standard normal variate (SNV); (D) first derivatives + standard normal variate (SNV).

Data noise and redundant spectral information must be removed to develop an acceptable prediction model based on spectral data and to obtain better prediction results. Hence, we preprocessed data for the UBC, PBC, and OBC sample groups using SNV and MSC. Preprocessing using Golay's derivative method was also performed to ensure the reliability of the models used in this study (Fig. 4). To avoid the influence caused by measurement environment and deficient system components, acquired HSI data were handled by different spectral preprocessing methods (Fig. 4). Spectral data (Fig. 4) handled by using different preprocessing methods shows (A) first derivatives + multiple scattering corrections (MSC); (B) second derivatives + multiple scattering corrections (MSC); (C) first derivatives + standard normal variate (SNV); (D) first derivatives + standard normal variate (SNV). After preprocessing treatment, the spectral data retained the absorption characteristics of the raw spectrum and improved in varying degrees. MSC and SNV could effectively remove the errors caused by spectral scattering in the spectral data, and MSC had a better ability to correct the spectral data than SNV. In addition, the following common machine learning algorithms were applied to achieve a predictive model for the classification of the salinity of brined kimchi cabbage: MLP, RF, XGBoost, K-NN, NBC, and DT (Table 1). Table 1 summarizes the performance evaluation using the test sets and the results of cross-validation with the training sets in terms of accuracy, specificity, F1 score, precision, and recall. The MLP, RF, XGBoost, K-NN, NBC, and in total) were established based on the full wavelengths following preprocessing. The best prediction results were obtained when the pretreatment was combined with MSC and Savitzky–Golay first derivative was combined with six models. In addition to accuracy, recall is an important evaluation index for model performance testing. In particular, as the purpose of the prediction model is to determine QUGs accurately and contribute to improving the quality of kimchi through early classification, accurate prediction of the quality of kimchi without missing QUGs is crucial. Therefore, we considered the probability of predicting the actual QUG as higher, so that a prediction model with better recall would exhibit superior performance. Precision is an extremely important evaluation indicator for quality control. This is because if the model incorrectly classifies QAG as QUG and removes acceptable brined kimchi cabbage samples, it would incur economic losses. In conclusion, the most reasonable approach is the use of precision, recall, and accuracy complementarily such that the model is effective when the three evaluation indicators are higher. Overall, the DT model based on the combined MSC and Savitzky–Golay first derivative preprocessing was determined as the best model.

**Table 1:** Results of cross validation with training sets of predictive models for classification the brining level according to salinity level of brined kimchi cabbage.

Models	Pre-treatment	Accuracy	Specificity	F1	Precision	Recall
MLP	MSC_1st-Der.	0.81	0.75	0.82	0.88	0.81
	MSC_2nd-Der.	0.60	0.41	0.61	0.61	0.65
	SNV_1st-Der.	0.70	0.51	0.68	0.72	0.68
	SNV_2nd-Der.	0.68	0.47	0.67	0.72	0.65
RF	**MSC_1st-Der.**	**0.89**	**0.82**	**0.88**	**0.91**	**0.85**
	MSC_2nd-Der.	0.75	0.57	0.73	0.82	0.69
	SNV_1st-Der.	0.81	0.69	0.82	0.83	0.79
	SNV_2nd-Der.	0.72	0.53	0.70	0.81	0.66
XGBoost	**MSC_1st-Der.**	**0.88**	**0.81**	**0.87**	**0.90**	**0.86**
	MSC_2nd-Der.	0.58	0.31	0.55	0.56	0.54
	SNV_1st-Der.	0.80	0.67	0.79	0.81	0.77
	SNV_2nd-Der.	0.60	0.35	0.57	0.58	0.56
K-NN	MSC_1st-Der.	0.87	0.79	0.86	0.89	0.84
	MSC_2nd-Der.	0.70	0.54	0.70	0.74	0.72
	SNV_1st-Der.	0.79	0.65	0.71	0.79	0.76
	SNV_2nd-Der.	0.72	0.57	0.72	0.75	0.73
NBC	MSC_1st-Der.	0.83	0.73	0.83	0.83	0.84
	MSC_2nd-Der.	0.75	0.58	0.74	0.77	0.72
	SNV_1st-Der.	0.70	0.53	0.70	0.71	0.70
	SNV_2nd-Der.	0.77	0.58	0.74	0.77	0.72
DT	**MSC_1st-Der.**	**0.93**	**0.90**	**0.94**	**0.93**	**0.95**
	MSC_2nd-Der.	0.60	0.41	0.61	0.61	0.65
	SNV_1st-Der.	0.76	0.57	0.73	0.82	0.69
	SNV_2nd-Der.	0.58	0.31	0.54	0.56	0.54

MLP: Multi-Layer Perceptron; RF: Randome forests; XGBoost: eXtreme Gradient Boosting; K-NN: K-Nearest Neighbors; NBC: Naive Bayes classifier; DT: Decision Tree; MSC: Multiplicative Scatter Correction; Der.: Derivative; SNV: Standard Normal Variate.

### 3.4 Classification models based on the selected wavelengths

Several wavelengths that conveyed the most important information and represented the entire spectrum were selected and classified as the optimal wavelengths. The variable importance in projection (VIP) scores was obtained (Table 2) using the BigZami software to reduce the number of wavelengths in the model classifier and improve the accuracy of the real-time classification model. Among the 24 models, the optimal wavelength was selected for three models (RF, XGBoost, and DT) based on the accuracy, precision, and recall. The number of variables was reduced from 195 to 10 or 11 wavelengths in the hyperspectral image range based on the VIP scores (Table 2 and Fig. S1). The source code of RF, XGBoost, and DT model were provided to validate for scientific quality in Fig. S2–4, respectively. Compared with the number of full spectral bands, the number of selected wavelengths decreased by 94.3 % for the water content. As shown in Table 2, the largest number of important wavelengths associated with water content was presented using the RF model following MSC_1st-derived preprocessing to classify the QUG efficiently. Water content and sensitivity are key metrics for evaluating osmotic action in brined kimchi cabbages. The wavelengths of the hyperspectral image range, which are related to moisture absorption, moisture sensitivity, and internal chemical composition (combination bands of O–H) were 935, 980, 1062, 1180, 1200, 1238, and 1245 nm (Table S2) [25,26,28,29].

**Table 2:** Significant wavelengths by the classification model, which efficiently classifies salinity level in brined kimchi cabbage.

Pre-treatment	Models	Optimal wavelengths (nm)
MSC_1st-Der.	RF	**935**, 971, **980**, 1076, 1118, **1238**, **1245**, 1259, 1266, 1709
	XGBoost	**935**, 971, **980**, **1238**, **1245**, 1498, 1632, 1646, 1653, 1660
	DT	**935**, 964, **980**, **1062**, 1147, **1180**, **1200**, **1238**, **1245**, 1252, 1674

RF: Randome forests; XGBoost: eXtreme Gradient Boosting; DT: Decision Tree; MSC: Multiplicative Scatter Correction; Der.: Derivative.

^1)^ Bold letters indicate wavelengths closely related to moisture absorption and moisture sensitivity.

### 3.5 Visualization of the salinity of brined kimchi cabbage

**Fig. 5:**
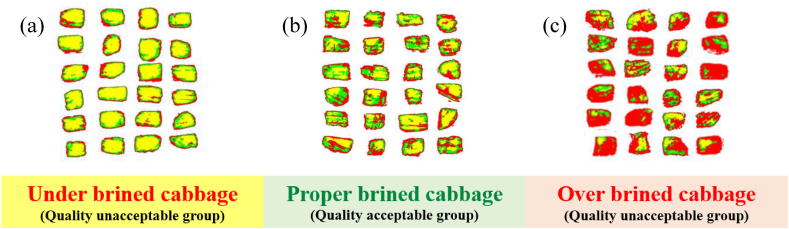
RGB images of the (a) UBC, (b) PBC, and (c) OBC samples.

A successful model using hyperspectral image can determine the internal composition of a target material in a rapid and non-destructive manner as well as visualize the spatial distribution of the binding bands between the chemical components of each target material. The raw hyperspectral images and corresponding visualization maps of brined kimchi cabbage samples for the water content concentrations predicted using the ELM model coupled with the SPA method are shown in Fig. 5, where the mass transfer of water and salt solids by osmosis occurred. The color scale is related to the moisture content concentration owing to moisture escape and solid salt infiltration. The color changes of the sample from yellow to green and red suggest a decrease in moisture concentration. As shown in Fig. 5(b), properly brined kimchi cabbage pieces (with salinities of more than 1 % and less than 1.8 %) were harmoniously distributed in yellow, green, and red, which explains the yellow and red colors in Fig. 5(a) and (c), respectively. Such visualization data are valuable for the real-time monitoring of the salinity level of brined kimchi cabbage during mass kimchi production.

## 4 Conclusions

In this study, chemometric tools based on hyperspectral imaging spectra (874–1734 nm) were used to classify the salinity grades of brined kimchi cabbage rapidly and non-the moisture content based on osmosis was set as a reference to evaluate and classify the salinity grade of brined kimchi cabbage that cannot be accurately observed by the naked eye. The experimental results showed that the DT model combined MSC and Savitzky–Golay first derivative preprocessing was most effective for classifying salinity grades based on moisture loss. Accuracy, precision, and recall rate were all larger than 90 % in the DT model. In addition, we investigated the feasibility of the salinity grade monitoring system for brined kimchi cabbage based on hyperspectral imaging analysis at specific optimized wavelengths (935, 964, 980, 1062, 1147, 1180, 1200, 1238, 1245, 1252, and 1674 nm) as suggested by this model. Our findings suggest that this multispectral imaging technique is applicable for the quality control of raw and subsidiary materials during the industrial kimchi manufacturing process. Furthermore, based on this research data, it could prove beneficial for enhancing real time quality analysis in the kimchi cabbage brining production process.

## CRediT authorship contribution statement

**Hyeyeon Song:** Writing – original draft, Visualization, Validation, Software, Methodology, Investigation, Data curation, Conceptualization. **Myounghwan Kim:** Formal analysis, Conceptualization. **Kwang Sun Yoo:** Resources, Data curation, Conceptualization. **Ji-Hyoung Ha:** Writing – original draft, Visualization, Validation, Project administration, Funding acquisition, Data curation, Conceptualization.

## Data availability statement

Data will be made available on request.

## Funding

This work was supported by the 10.13039/501100003722World Institute of Kimchi (grant numbers KE2302-2 and KE2402-2) funded by the Ministry of Science, ICT, and Future Planning, Republic of Korea.

## Declaration of competing interest

The authors declare that they have no known competing financial interests or personal relationships that could have appeared to influence the work reported in this paper.
