# Supplementary material for: Rapid and non-destructive classification of salinity levels in brined kimchi cabbage using hyperspectral imaging
Source: Heliyon. 2024 Nov 29;10(23):e40817. doi: 10.1016/j.heliyon.2024.e40817 (PMC11648222; doi:10.1016/j.heliyon.2024.e40817)
Supplement: Multimedia component 1 [file mmc1.docx]

**Rapid and non-destructive classification of salinity levels in brined kimchi cabbage using hyperspectral imaging**

**Hyeyeon Song^a^, Myounghwan Kim^b^, Kwang Sun Yoo^b^, and Ji-Hyoung Ha^a,^***

^a^ Hygienic Safety and Materials Research Group, World Institute of Kimchi, 86 Kimchi-ro, Nam-gu, Gwangju, Republic of Korea

^b^ AI Research, Elroilab Co., Ltd., 28 Digital-ro 30-gil, Guro-gu, Seoul, Republic of Korea

* Corresponding author. E-mail: [hajee@wikim.re.kr](mailto:hajee@wikim.re.kr); Tel.: 82-62-610-1845; Fax: 82-62-610-1810

**Figure legends**

**Fig. S1.** Significant wavelengths by three models (a) RF, (b) XGBoost, and (c) DT.

**Fig. S2.** Randome forests source code

**Fig. S3.** eXtreme Gradient Boosting source code

**Fig. S4.** Decision Tree source code

Table S1. Statistics results of salinity and water contents in calibration and independent test set.

|  | Data set | Sample number | Range (%) | Mean (%) | Standard Deviation |
| --- | --- | --- | --- | --- | --- |
| Salinity | Calibration set | 135 | 0.68-2.32 | 1.45 | 0.62 |
|  | Prediction set | 45 | 0.61-2.97 | 1.47 | 0.67 |
| Water content | Calibration set | 135 | 89.18-94.29 | 92.09 | 1.44 |
|  | Prediction set | 45 | 90.01-95.15 | 91.98 | 1.23 |

Table S2. Speculative assignments of absorption bands in peak intensity in the average patterns of hyperspectral wavelength (nm).

| Wavelength (nm) | Importance and physical significance of wavelength index in vegetation and cropland studies | References |
| --- | --- | --- |
| **935^1)^** | Water (water sensitivity), Moisture, Biomass, Leaf water | Thenkabail et al., 2014 |
| **980** | Water (water sensitivity), Moisture, Leaf water | Paul, 1989 |
| **1062** | Water content | Sun et al. (2018) |
| 1070 | Total chlorophyll, Anthocyanin, and Carotenoids | Prasad et al., 2014 |
| 1108 | Water content | Sun et al. (2018) |
| **1180** | Water (water sensitivity) | Prasad et al., 2014 |
| **1200** | Water (water sensitivity), Lignin, Cellulose, Starch | Prasad et al., 2014 |
| **1238** | Water (water sensitivity), Leaf water, Biomass | Paul, 1989 |
| **1245** | Water (water sensitivity) | Esquerre et al. (2012) |
| **1430** | Water (water sensitivity), Moisture, Lignin, and Cellulose | Paul, 1989, Sun et al. (2018) |
| 1490 | Cellulose | Prasad et al., 2014, Paul, 1989 |
| **1518** | Moisture and Biomass | Paul, 1989 |
| 1530 | Starch | Prasad et al., 2014 |
| 1540 | Cellulose, Starch | Paul, 1989 |
| 1580 | Starch | Paul, 1989 |
| **1620** | Moisture | Paul, 1989 |
| **1650** | Heavy metal stress, Moisture, and Chlorophyll | Prasad et al., 2014 |
| 1690 | Lignin, Starch | Prasad et al., 2014 |

^1)^ Bold letters indicate wavelengths closely related to moisture absorption and moisture sensitivity.

Fig. S1.

| (a)  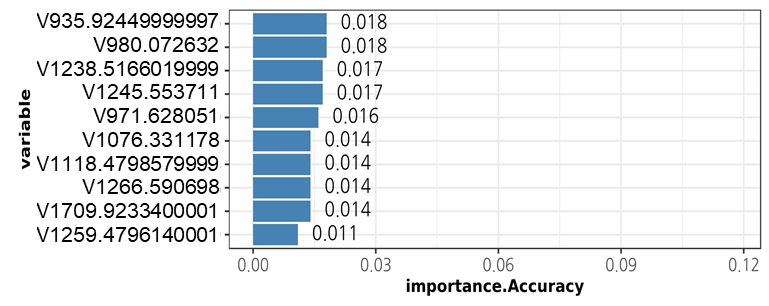 |
| --- |
| (b)  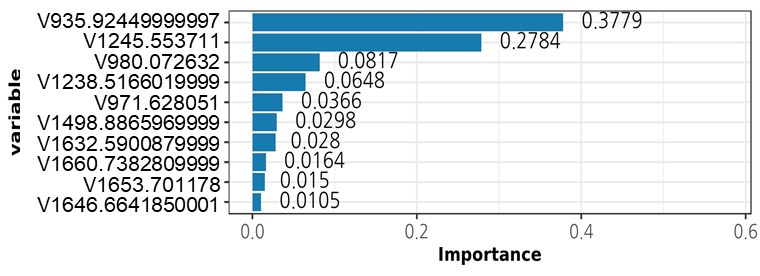 |
| (c)  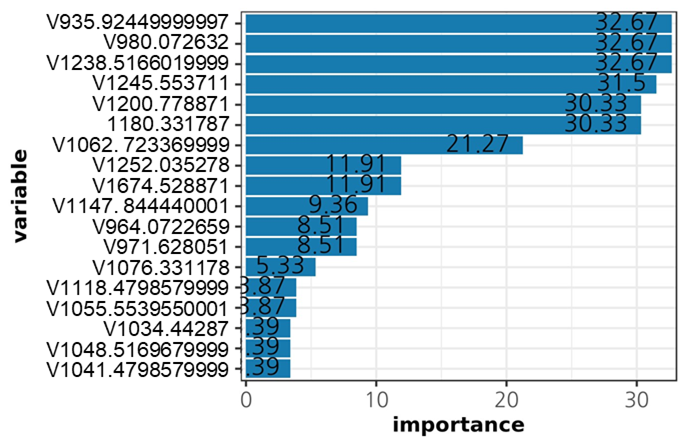 |

Fig. S2.

| ############################### Random Forest ####################################  #################### "Model Summary, Validation Metrics" ########################  df <- read.csv("C:/Users/user/Downloads/filename.csv")  xvar <- colnames(df)[c()]  yvar <- colnames(df)[c()]  mtry <- 2  ntree <- 50  train_ratio <- 70  validation_ratio <- 30  seedNum <- 1234  nodesize=5  maxnodes=  sampsize=71  library(randomForest)  if(sum(is.na(df[xvar])) > 0) {  stop("[0009] NA exists.", call. = F)  }  #hyper-parameter  if(nodesize == 'nodesize'){  nodesize = 5 #Classification is 1, Regression is 5  }  if(sampsize == 'sampsize'){  sampsize = ceiling(0.632*nrow(df))  }  if(maxnodes == ' '){  maxnodes = NULL  }  # Splitting the Training Data and Validation Data  set.seed(seedNum)  ratio_sum <- sum(train_ratio, validation_ratio)  train_ratio <- train_ratio / ratio_sum  smp_size <- floor(nrow(df) *(train_ratio))  train_idx <- sample(seq_len(nrow(df)), size = smp_size)  train <- df[train_idx, ] # Training Data  validation <- df[-train_idx, ] # Validation Data  if(train_ratio == 1) {  # Data used for training the model with training data only  validation_x <- df[xvar]  validation_y <- df[[yvar]]  } else {  # Data used for training the model with training and validation data  validation_x <- validation[xvar]  validation_y <- validation[[yvar]]  }  # Independent variables for R-squared calculation  r2_xvar <- xvar  # Create a random forest model  xvar <- paste0(xvar, collapse = "+")  cmd <- paste0("rf.fit <- randomForest(", yvar, " ~", xvar, ",data = train", ", mtry =", mtry,  ", ntree =", ntree , ", importance=T, nodesize = ", nodesize,", sampsize=", sampsize,", maxnodes=", maxnodes,")")  eval(parse(text=cmd))  # Generate predictions based on the created model  y_pred <- predict(rf.fit, validation_x)  #Predicted values to be used for calculating R-squared  r2_pred <- predict(rf.fit, train[r2_xvar])  # Extract the OOB error value  oob_error <- rf.fit$mse[length(rf.fit$mse)]  # Calculate model evaluation metrics  mse <- mean((validation_y-y_pred)^2)  rmse <- sqrt(mse)  mae <- mean(abs(validation_y-y_pred))  mape <- mean(abs((validation_y - y_pred)/validation_y))  R2 <- 1-(sum((train[[yvar]]-r2_pred)^2)/sum((train[[yvar]]-mean(train[[yvar]]))^2))  n <- nrow(train)  k <- length(r2_xvar)  adj_R2 <- 1-((1-R2)*(n-1)/(n-k-1))  model_stat <- data.frame(OOB_error = oob_error, MSE = mse, RMSE = rmse, MAE = mae, MAPE = mape, R2 = R2, Adj_R2 = adj_R2)  model_stat <- round(model_stat, 4)  # model summary information  model_summary <- capture.output(rf.fit)  model_summary[length(model_summary)+1] <- ""  model_summary[length(model_summary)+1] <- paste0("OOB error: ", oob_error)  model_summary[length(model_summary)+1] <- paste0("MSE(validation_y, y_pred): ", mse)  model_summary[length(model_summary)+1] <- paste0("RMSE(validation_y, y_pred): ", rmse)  model_summary[length(model_summary)+1] <- paste0("MAE: ", mae)  model_summary[length(model_summary)+1] <- paste0("MAPE(%): ", mape * 100, "%")  model_summary[length(model_summary)+1] <- ""  # Create a table containing actual and predicted values  df_val_pred <- data.frame(validation_y, y_pred)  colnames(df_val_pred) <- gsub("validation_y", "actual_y", colnames(df_val_pred))  # Generate results in the form of a list  result <- list(model = rf.fit, model_summary = model_summary, model_stat = model_stat,  df_val_pred = df_val_pred)  #################### "Feature importance graph" ########################  library(ggplot2)  library(gridExtra)  # Use only the model information from the results generated in the form of a list  model <- result$model  importance <- result$model$importance  importance <- as.data.frame(importance)  importance.MSE <- importance$`%IncMSE`  importance.MSE <- as.data.frame(importance.MSE)  row.names(importance.MSE) <- row.names(importance)  importance.Pur <- importance$IncNodePurity  importance.Pur <- as.data.frame(importance.Pur)  variable <- row.names(importance.MSE)  # Create a table for the feature importance graph  df1 <- data.frame(variable = variable, importance = round(importance.MSE, 2))  df2 <- data.frame(variable = variable, importance = round(importance.Pur, 2))  # Plot the feature importance graph  image1 <- ggplot(df1, aes(x=reorder(variable, importance.MSE), y= importance.MSE)) +  geom_bar(stat = "identity", fill = "steelblue") +  scale_y_continuous(limits = c(0,max(importance.MSE)+0.2)) +  geom_text(aes(label = importance.MSE), hjust = -0.3, vjust = 0.3, size = 4) +  theme_minimal() +  coord_flip() +  xlab("variable")  image2 <- ggplot(df2, aes(x=reorder(variable, importance.Pur), y= importance.Pur)) +  geom_bar(stat = "identity", fill = "steelblue") +  scale_y_continuous(limits = c(0,max(importance.Pur)+5)) +  geom_text(aes(label = importance.Pur), hjust = -0.3, vjust = 0.3, size = 4) +  theme_minimal() +  coord_flip() +  xlab("variable")  grid.arrange(image1, image2, ncol=1)  #################### "Error graph" ########################  library(ggplot2)  # Use only the model information from the results generated in list form  ntree <- result$model$ntree  mse <- result$model$mse  # Create a table for the OOB error graph  OOB_tmp <- data.frame(Value = mse, Index = 1:ntree)  # Plot the OOB error graph  ggplot(data=OOB_tmp, aes(x=Index, y=Value)) + geom_line(size = 1) +  geom_point(size = 0.6) + labs(x="Number of tree", y="OOB error(%)") + ggtitle("Out of Bag Error")  #################### "Result graph" ########################  library(ggplot2)  # Use only the actual values and predicted values from the results generated in the form of a list  actual_y <- result$df_val_pred$actual_y  y_pred <- result$df_val_pred$y_pred  # Create a table for generating a line graph (actual values)  actual_y_tmp <- data.frame(Value = actual_y,  Class = rep("actual_y", length(actual_y)),  Index = 1:length(actual_y))  #Create a table for generating a line graph (predicted values)  y_pred_tmp <- data.frame(Value = y_pred,  Class = rep("y_pred", length(y_pred)),  Index = 1:length(y_pred))  # Merge the two tables  df <- rbind(actual_y_tmp, y_pred_tmp)  # Plot a line graph for actual and predicted values  ggplot(data=df, aes(x=Index, y=Value)) + geom_line(aes(color=Class), size=1)  ############################### Classification Model ####################################  #################### "Model Summary, Validation Metrics" ########################  df <- read.csv("C:/Users/user/Downloads/filename.csv")  xvar <- colnames(df)[c()]  yvar <- colnames(df)[c()]  mtry <- 2  ntree <- 50  train_ratio <- 70  validation_ratio <- 30  seedNum <- 1234  nodesize=5  maxnodes=  sampsize=71  library(randomForest)  library(caret)  library(pROC)  library(ggplot2)  if(sum(is.na(df[xvar])) > 0) {  stop("[0009] NA exists.", call. = F)  }  #hyper-parameter  if(nodesize == 'nodesize'){  nodesize = 5 #Classification is 1, Regression is 5  }  if(sampsize == 'sampsize'){  sampsize = ceiling(0.632*nrow(df))  }  if(maxnodes == ' '){  maxnodes = NULL  }  # Splitting the Training Data and Validation Data  set.seed(seedNum)  ratio_sum <- sum(train_ratio, validation_ratio)  train_ratio <- train_ratio / ratio_sum  # Perform stratified sampling for each class of the dependent variable  suppressWarnings(train_idx <- createDataPartition(df[[yvar]], p = train_ratio, list = F))  train <- df[train_idx, ] # Training Data  validation <- df[-train_idx, ] # Validation Data  if(train_ratio == 1) {  # Data used for training the model with training data only  validation_x <- df[xvar]  validation_y <- df[[yvar]]  } else {  # Data used for training the model with training and validation data  validation_x <- validation[xvar]  validation_y <- validation[[yvar]]  }  # Create a random forest classification model  xvar <- paste0(xvar, collapse = "+")  cmd <- paste0("rf.fit <- randomForest(", yvar, " ~", xvar, ",data = train", ", mtry =", mtry,  ", ntree =", ntree , ", importance=T, nodesize = ", nodesize,", sampsize=", sampsize,", maxnodes=", maxnodes,")")  eval(parse(text=cmd))  # Generate predictions based on the created model  y_pred <- predict(rf.fit, validation_x)  # Generate confusion matrix information  cm <- confusionMatrix(y_pred, validation_y)  # Create a model evaluation metrics table  Acc <- cm$overall[1]  Kap <- cm$overall[2]  model_stat <- data.frame(Accuracy = Acc, Kappa = Kap)  model_stat <- round(model_stat, 4)  row.names(model_stat) <- NULL  if(class(cm$byClass) != "matrix") {  # Binary classification  model_stat2 <- as.data.frame(t(cm$byClass[c("Sensitivity", "Specificity", "Precision", "Recall", "F1")]))  model_stat2 <- round(model_stat2, 4)  row.names(model_stat2) <- NULL    } else {  # Multiclass classification  model_stat2 <- as.data.frame(cm$byClass)[c("Sensitivity", "Specificity", "Precision", "Recall", "F1")]  model_stat2 <- round(model_stat2, 4)  classname <- sapply(strsplit(row.names(model_stat2), ":"), "[", 2)  model_stat2$Class <- classname  model_stat2 <- model_stat2[c(ncol(model_stat2), 1:(ncol(model_stat2)-1))]  row.names(model_stat2) <- NULL    }  # model summary information  model_summary <- capture.output(rf.fit, cm$overall)  # "Change the class to resolve the issue of missing values where TRUE/FALSE is required  y_pred <- as.character(y_pred)  validation_y <- as.character(validation_y)  # Create a table containing actual values, predicted values, and prediction probabilities  df_val_pred <- data.frame(validation_y, y_pred)  colnames(df_val_pred) <- gsub("validation_y", "actual_y", colnames(df_val_pred))  # Generate results in the form of a list  result <- list(model = rf.fit, model_summary = model_summary, model_stat = model_stat, model_stat2 = model_stat2,  validation_x = validation_x, validation_y = validation_y,  df_val_pred = df_val_pred, cm = cm)  #################### " feature importance graph" ########################  library(ggplot2)  library(gridExtra)  # Use only the model information from the results generated in list form  model <- result$model  importance <- result$model$importance  importance <- as.data.frame(importance)  importance.Acc <- importance$MeanDecreaseAccuracy  importance.Acc <- as.data.frame(importance.Acc)  row.names(importance.Acc) <- row.names(importance)  importance.Gini <- importance$MeanDecreaseGini  importance.Gini <- as.data.frame(importance.Gini)  variable <- row.names(importance.Acc)  # Create a table for the feature importance graph  df1 <- data.frame(variable = variable, importance = round(importance.Acc, 3))  df2 <- data.frame(variable = variable, importance = round(importance.Gini, 2))  # Plot the feature importance graph  image1 <- ggplot(df1, aes(x=reorder(variable, importance.Acc), y= importance.Acc)) +  geom_bar(stat = "identity", fill = "steelblue") +  scale_y_continuous(limits = c(0,max(importance.Acc)+0.1)) +  geom_text(aes(label = importance.Acc), hjust = -0.3, vjust = 0.3, size = 4)+  theme_minimal() +  coord_flip() +  xlab("variable")  image2 <- ggplot(df2, aes(x=reorder(variable, importance.Gini), y= importance.Gini)) +  geom_bar(stat = "identity", fill = "steelblue") +  scale_y_continuous(limits = c(0,max(importance.Gini)+5)) +  geom_text(aes(label = importance.Gini), hjust = -0.3, vjust = 0.3, size = 4)+  theme_minimal() +  coord_flip() +  xlab("variable")  grid.arrange(image1, image2, ncol=1)  #################### "error graph" ########################  library(ggplot2)  # Use only the model information from the results generated in list form  ntree <- result$model$ntree  err.rate <- result$model$err.rate[, 1]  # Create a table for the OOB error graph  OOB_tmp <- data.frame(Value = err.rate, Index = 1:ntree)  #Plot the OOB error graph  ggplot(data=OOB_tmp, aes(x=Index, y=Value)) + geom_line(size = 1) +  geom_point(size = 0.6) + labs(x="Number of tree", y="OOB error(%)") + ggtitle("Out of Bag Error")  #################### "Correlation matrix" ########################  library(ggplot2)  library(svglite)  library(plyr)  library(dplyr)  # Use only the actual values and predicted values from the results generated in the form of a list  actual_y <- result$df_val_pred$actual_y  y_pred <- result$df_val_pred$y_pred  # Generate confusion matrix information  confusionM <- xtabs(~y_pred + actual_y)  # Preprocessing for plotting the confusion matrix graph  input.matrix <- data.matrix(confusionM)  confusion <- as.data.frame(as.table(input.matrix))  colnames(confusion)[3] <- "Frequency"  # Plot the confusion matrix graph  ggplot(confusion, aes(x = actual_y, y = y_pred, fill = Frequency)) + geom_tile() +  geom_text(aes(label=confusion$Frequency)) +  scale_x_discrete(name="Actual Class") + scale_y_discrete(name="Predicted Class") +  geom_text(aes(label = Frequency),colour = "white") +  scale_fill_continuous(high = "#132B43", low = "#56B1F7") |
| --- |

**Fig. S3.**

| ############################### XGBoost ####################################  #################### "Model Summary, Validation Metrics" ########################  df <- read.csv("C:/Users/user/Downloads/filename.csv")  xvar <- colnames(df)[c(2, 3, 4, 5, 6, 7, 8, 9, 10, 11, 12, 13, 14, 15, 16, 17, 18, 19, 20, 21, 22, 23, 24, 25, 26, 27, 28, 29, 30, 31, 32, 33, 34, 35, 36, 37, 38, 39, 40, 41, 42, 43, 44, 45, 46, 47, 48, 49, 50, 51, 52, 53, 54, 55, 56, 57, 58, 59, 60, 61, 62, 63, 64, 65, 66, 67, 68, 69, 70, 71, 72, 73, 74, 75, 76, 77, 78, 79, 80, 81, 82, 83, 84, 85, 86, 87, 88, 89, 90, 91, 92, 93, 94, 95, 96, 97, 98, 99, 100, 101, 102, 103, 104, 105, 106, 107, 108, 109, 110, 111, 112, 113)]  yvar <- colnames(df)[c(115)]  eta <- c(0.3)  gamma <- c(0)  nrounds <- 50  subsample <- 1  maxdepth <- 6  train_ratio <- 70  validation_ratio <- 30  seedNum <- 1234  min_child_weight= 1  lambda = 0  lambda_bias = 0  alpha = 0  num_parallel_tree = 1  library(xgboost)  library(dplyr)  library(reshape2)  library(doParallel)  library(foreach)  # Splitting the Training Data and Validation Data  set.seed(seedNum)  ratio_sum <- sum(train_ratio, validation_ratio)  train_ratio <- train_ratio / ratio_sum  smp_size <- floor(nrow(df) *(train_ratio))  train_idx <- sample(seq_len(nrow(df)), size = smp_size)  train <- df[train_idx, ] # Training Data  validation <- df[-train_idx, ] # Validation Data  if(train_ratio == 1) {  # Data used for training the model with training data only  validation_x <- df[xvar] %>% data.matrix() # Convert to matrix format  validation_y <- df[[yvar]]  } else {  # Data used for training the model with training and validation data  validation_x <- validation[xvar] %>% data.matrix() # Convert to matrix format  validation_y <- validation[[yvar]]  }  # Convert the training data into matrix form  train_x <- train[xvar] %>% data.matrix()  train_y <- train[[yvar]]  # Grid search parallel processing  grid <- expand.grid(eta = eta, gamma = gamma)  model <- list()  grid_search <- list()  #Create a model  model <-xgb.cv(data = train_x, label = train_y,  nfold = 5, nrounds = nrounds,  early_stopping_rounds = ifelse(nrounds == 1, 1, round(nrounds/2)), # about folds and rounds  subsample = subsample, max_depth = maxdepth,  objective = 'reg:linear', verbose = F,  params = grid[1:nrow(grid),])  #Save the optimal value  grid_search <- data.frame(train_rmse_last = unlist(model$evaluation_log[,2]) %>% last,  valid_rmse_last = unlist(model$evaluation_log[,4]) %>% last)  #Save performance for each round  nround_er_df=data.frame(error = c(unlist(model$evaluation_log[,2]), unlist(model$evaluation_log[,4])),  class = c(rep('train', nrow(model$evaluation_log)),  rep('validation', nrow(model$evaluation_log))),  nrounds = rep(1:nrow(model$evaluation_log)))  #Modeling with XGBoost using optimal parameters  model <- xgboost(data = train_x, label = train_y,  nfold = 5, nrounds = nrounds,  early_stopping_rounds = ifelse(nrounds == 1, 1, round(nrounds/2)),  subsample = subsample, max_depth = maxdepth,  objective = 'reg:linear', verbose = F,  params = grid[which.min(grid_search$valid_rmse_last),],  min_child_weight = min_child_weight, lambda = lambda, alpha = alpha,  num_parallel_tree = num_parallel_tree)  model_summary <- capture.output(model)[4:9]  # Output the predicted values  y_pred <- predict(model, validation_x)  # Output the predicted values to be used for calculating R2  r2_pred <- predict(model, train_x)  # Calculate model evaluation metrics  mse <- base::mean((validation_y-y_pred)^2)  rmse <- sqrt(mse)  mae <- base::mean(abs(validation_y-y_pred))  mape <- base::mean(abs((validation_y - y_pred)/validation_y))  R2 <- 1-(base::sum((train_y-r2_pred)^2)/base::sum((train_y-base::mean(train_y))^2))  n <- nrow(train)  k <- length(xvar)  adj_R2 <- 1-((1-R2)*(n-1)/(n-k-1))  model_stat <- data.frame(MSE = mse, RMSE = rmse, MAE = mae, MAPE = mape, R2 = R2, Adj_R2 = adj_R2)  model_stat <- round(model_stat, 4)  # Create a table containing actual and predicted values  df_val_pred <- data.frame(validation_y, y_pred)  colnames(df_val_pred) <- gsub("validation_y", "actual_y", colnames(df_val_pred))  result <- list(model = model, model_summary = model_summary, model_stat = model_stat, df_val_pred = df_val_pred,  grid = grid, grid_search = grid_search, nround_er_df = nround_er_df)  result  #################### "CV graph" ########################  library(ggplot2)  #Use only the necessary information from the results generated in list form  nround_er_df=result$nround_er_df  #Graph  ggplot(data=nround_er_df,aes(x=nrounds,y=error,col=class))+  geom_point(alpha=0.2)+  geom_smooth(alpha = 0.4, se = F)+  theme_bw()+scale_color_manual(values = c('#4682B4','#FF797A','#FFCB62'))+  theme(#title = element_text(size = 25, face = "Bold")  axis.title.x = element_text(size=10, face = 'bold'), #Axis labels  axis.title.y = element_text(size=10, face = 'bold'),  axis.text.x = element_text(size=10),  axis.text.y = element_text(size =10),  legend.text = element_text(size=10),  legend.title = element_text(size=10, face="bold"),  legend.position = "right"  )  #################### "Feature importance graph" ########################  library(Ckmeans.1d.dp)  model <- result$model  # Create a feature importance dataframe  imp <- xgb.importance(model = model)  # graph  xgb.ggplot.importance(imp)  #################### "Result Graph" ########################  # Use only the actual values and predicted values from the results generated in the form of a list  actual_y <- result$df_val_pred$actual_y  y_pred <- result$df_val_pred$y_pred  #Create a table for generating a line graph (actual values)  actual_y_tmp <- data.frame(Value = actual_y,  Class = rep("actual_y", length(actual_y)),  Index = 1:length(actual_y))  # Create a table for generating a line graph (predicted values)  y_pred_tmp <- data.frame(Value = y_pred,  Class = rep("y_pred", length(y_pred)),  Index = 1:length(y_pred))  # Merge the two tables  df <- rbind(actual_y_tmp, y_pred_tmp)  # Plot a line graph for actual and predicted values  ggplot(data=df, aes(x=Index, y=Value)) + geom_line(aes(color=Class), size=1)  ############################### Classification Model ####################################  #################### " Model Summary, Validation Metrics" ########################  df <- read.csv("C:/Users/user/Downloads/filename.csv")  xvar <- colnames(df)[c(2, 3, 4, 5, 6, 7, 8, 9, 10, 11, 12, 13, 14, 15, 16, 17, 18, 19, 20, 21, 22, 23, 24, 25, 26, 27, 28, 29, 30, 31, 32, 33, 34, 35, 36, 37, 38, 39, 40, 41, 42, 43, 44, 45, 46, 47, 48, 49, 50, 51, 52, 53, 54, 55, 56, 57, 58, 59, 60, 61, 62, 63, 64, 65, 66, 67, 68, 69, 70, 71, 72, 73, 74, 75, 76, 77, 78, 79, 80, 81, 82, 83, 84, 85, 86, 87, 88, 89, 90, 91, 92, 93, 94, 95, 96, 97, 98, 99, 100, 101, 102, 103, 104, 105, 106, 107, 108, 109, 110, 111, 112, 113)]  yvar <- colnames(df)[c(115)]  eta <- c(0.3)  gamma <- c(0)  nrounds <- 50  subsample <- 1  maxdepth <- 6  train_ratio <- 70  validation_ratio <- 30  seedNum <- 1234  min_child_weight= 1  lambda = 0  lambda_bias = 0  alpha = 0  num_parallel_tree = 1  library(xgboost)  library(dplyr)  library(caret)  library(reshape2)  library(doParallel)  library(foreach)  #Splitting the Training Data and Validation Data  set.seed(seedNum)  ratio_sum <- sum(train_ratio, validation_ratio)  train_ratio <- train_ratio / ratio_sum  # yvar Stratified sampling  suppressWarnings(train_idx <- createDataPartition(df[[yvar]], p = train_ratio, list = F))  train <- df[train_idx, ] # Training Data  validation <- df[-train_idx, ] # Validation Data  if(train_ratio == 1) {  # Validation data to use when training the model with training data only  validation_x <- df[xvar] %>% data.matrix() # Convert to matrix form  validation_y <- df[[yvar]]  } else {  # Validation data to use when training with both training and validation sets  validation_x <- validation[xvar] %>% data.matrix() # Convert to matrix form  validation_y <- validation[[yvar]]  }  # Convert the training data into matrix form  train_x <- train[xvar] %>% data.matrix()  train_y <- train[[yvar]]  # Grid search parallel processing  grid <- expand.grid(eta = eta, gamma = gamma)  model <- list()  grid_search <- list()  # Create a model  model <- xgb.cv(data = train_x, label = as.numeric(train_y)-1,  num_class = levels(train_y) %>% length,  nfold = 5, nrounds = nrounds,  early_stopping_rounds = ifelse(nrounds == 1, 1, round(nrounds/2)), # about folds and rounds  subsample = subsample, max_depth = maxdepth,  objective = 'multi:softprob', verbose = F,  params = grid[1:nrow(grid),])  # Save the optimal value  grid_search <- data.frame(train_error_last = unlist(model$evaluation_log[,2]) %>% last,  valid_error_last = unlist(model$evaluation_log[,4]) %>% last)  #Save performance for each round  nround_er_df=data.frame(error = c(unlist(model$evaluation_log[,2]), unlist(model$evaluation_log[,4])),  class = c(rep('train', nrow(model$evaluation_log)),  rep('validation', nrow(model$evaluation_log))),  nrounds = rep(1:nrow(model$evaluation_log)))  # Modeling with XGBoost using optimal parameters  model <- xgboost(data = train_x, label = as.numeric(train_y)-1, num_class = levels(train_y) %>% length,  nfold = 5, nrounds = nrounds,  early_stopping_rounds = ifelse(nrounds == 1, 1, round(nrounds/2)),  subsample = subsample, max_depth = maxdepth,  objective = 'multi:softprob', verbose = F,  params = grid[which.min(grid_search$valid_error_last),],  min_child_weight = min_child_weight, lambda = lambda, alpha = alpha,  num_parallel_tree = num_parallel_tree)  # Output the model equation  model_summary <- capture.output(model)[4:9]  # Generate predictions based on the trained model  y_pred <- predict(model, validation_x) %>% matrix(ncol = levels(validation_y) %>% length, byrow=TRUE) %>%  as.data.frame() %>% mutate(pred = levels(validation_y)[max.col(.)] %>% as.factor()) %>%  select(pred) %>% .[[1]]  # confusion matrix  cm <- confusionMatrix(y_pred, validation_y)  # Accuracy, Kappa value  Acc <- cm$overall[1]  Kap <- cm$overall[2]  #Create a model evaluation metrics table  model_stat <- data.frame(Accuracy = Acc, Kappa = Kap)  model_stat <- round(model_stat, 4)  row.names(model_stat) <- NULL  if(class(cm$byClass) != "matrix") {  model_stat2 <- as.data.frame(t(cm$byClass[c("Sensitivity", "Specificity", "Precision", "Recall", "F1")]))  model_stat2 <- round(model_stat2, 4)  row.names(model_stat2) <- NULL  } else {  model_stat2 <- as.data.frame(cm$byClass)[c("Sensitivity", "Specificity", "Precision", "Recall", "F1")]  model_stat2 <- round(model_stat2, 4)  classname <- sapply(strsplit(row.names(model_stat2), ":"), "[", 2)  model_stat2$Class <- classname  model_stat2 <- model_stat2[c(ncol(model_stat2), 1:(ncol(model_stat2)-1))]  row.names(model_stat2) <- NULL  }  # "Change the class to resolve the issue of missing values where TRUE/FALSE is required  y_pred <- as.character(y_pred)  validation_y <- as.character(validation_y)  # Create a table containing actual values, predicted values, and prediction probabilities  df_val_pred <- data.frame(validation_y, y_pred)  colnames(df_val_pred) <- gsub("validation_y", "actual_y", colnames(df_val_pred))  result <- list(model = model, model_summary = model_summary, model_stat = model_stat, model_stat2 = model_stat2, df_val_pred = df_val_pred,  grid = grid, grid_search = grid_search, yvar = yvar, nround_er_df = nround_er_df)  result  #################### "CV graph" ########################  library(ggplot2)  #Use only the necessary information from the results generated in list form  nround_er_df=result$nround_er_df  #graph  ggplot(data=nround_er_df,aes(x=nrounds,y=error,col=class))+  geom_point(alpha=0.2)+  geom_smooth(alpha = 0.4, se = F)+  theme_bw()+scale_color_manual(values = c('#4682B4','#FF797A','#FFCB62'))+  theme(#title = element_text(size = 25, face = "Bold")  axis.title.x = element_text(size=10, face = 'bold'), #Axis labels  axis.title.y = element_text(size=10, face = 'bold'),  axis.text.x = element_text(size=10),  axis.text.y = element_text(size =10),  legend.text = element_text(size=10),  legend.title = element_text(size=10, face="bold"),  legend.position = "right"  )  #################### "Feature importance graph" ########################  library(Ckmeans.1d.dp)  model <- result$model  # Create a feature importance dataframe  imp <- xgb.importance(model = model)  # graph  xgb.ggplot.importance(imp)  #################### "confusion matrix graph" ########################  # Use only the actual values and predicted values from the results generated in the form of a list  actual_y <- result$df_val_pred$actual_y  y_pred <- result$df_val_pred$y_pred  df_y_unique <- unique(y_pred)  df_y_unique <- as.factor(df_y_unique)  validation_y_vec <- actual_y  validation_y_vec <- as.factor(validation_y_vec)  # Generate confusion matrix information  confusionM <- xtabs(~y_pred + actual_y)  # Preprocessing for plotting the confusion matrix graph  input.matrix <- data.matrix(confusionM)  confusion <- as.data.frame(as.table(input.matrix))  colnames(confusion)[3] <- "Frequency"  # graph  ggplot(confusion, aes(x = actual_y, y = y_pred, fill = Frequency)) + geom_tile() +  geom_text(aes(label=confusion$Frequency)) +  scale_x_discrete(name="Actual Class") + scale_y_discrete(name="Predicted Class") +  geom_text(aes(label = Frequency),colour = "white") +  scale_fill_continuous(high = "#132B43", low = "#56B1F7") |
| --- |

**Fig. S4**

| ############################### Decision Tree ####################################  #################### " Model Summary, Validation Metrics, CP Table" ########################  df <- read.csv("C:/Users/user/Downloads/filename.csv")  xvar <- colnames(df)[c()]  yvar <- colnames(df)[c()]  minbucket = 7  maxdepth = 2  train_ratio <- 70  validation_ratio <- 30  seedNum <- 1234  library(rpart)  # Splitting the Training Data and Validation Data  set.seed(seedNum)  ratio_sum <- sum(train_ratio, validation_ratio)  train_ratio <- train_ratio / ratio_sum  smp_size <- floor(nrow(df) *(train_ratio))  train_idx <- sample(seq_len(nrow(df)), size = smp_size)  train <- df[train_idx, ] # Training Data  validation <- df[-train_idx, ] # Validation Data  if(train_ratio == 1) {  # Data used for training the model with training data only  validation_x <- df[xvar]  validation_y <- df[[yvar]]  } else {  # Data used for training the model with training and validation data  validation_x <- validation[xvar]  validation_y <- validation[[yvar]]  }  # Specify minbucket, maxdepth  control <- rpart.control(minbucket = minbucket, maxdepth = maxdepth)  # Independent variables for R-squared calculation  r2_xvar <- xvar  # Create a decision tree regression model  xvar <- paste0(xvar, collapse = "+")  cmd <- paste0("m <- rpart(", yvar, " ~ ", xvar, ", data = train, control = ", control,")")  eval(parse(text=cmd))  # Check the appropriate number of terminal nodes for pruning  cp_check <- m$cptable  cp_check <- round(as.data.frame(cp_check), 8)  colnames(cp_check) <- c("Complexity(CP)", "Number of splits", "Error rate", "Cross-validation error", "Standard deviation of cross-validation error")  # Generate predictions based on the created model  y_pred <- predict(m, validation_x)  # Predicted values to be used for calculating R-squared  r2_pred <- predict(m, train[r2_xvar])  # Calculate model evaluation metrics  mse <- round(mean((validation_y-y_pred)^2), 3)  rmse <- round(sqrt(mse), 3)  mae <- round(mean(abs(validation_y-y_pred)), 3)  mape <- round(mean(abs((validation_y - y_pred)/validation_y))*100, 3)  R2 <- 1-(sum((train[[yvar]]-r2_pred)^2)/sum((train[[yvar]]-mean(train[[yvar]]))^2))  R2 <- round(R2, 3)  n <- nrow(train)  k <- length(r2_xvar)  adj_R2 <- 1-((1-R2)*(n-1)/(n-k-1))  adj_R2 <- round(adj_R2, 3)  # model summary information  model_summary <- capture.output(m)  model_summary[length(model_summary)+1] <- ""  model_summary[length(model_summary)+1] <- paste0("MSE: ", mse)  model_summary[length(model_summary)+1] <- paste0("RMSE: ", rmse)  model_summary[length(model_summary)+1] <- paste0("MAE: ", mae)  model_summary[length(model_summary)+1] <- paste0("MAPE(%): ", mape, "%")  model_summary[length(model_summary)+1] <- paste0("R2: ", R2)  model_summary[length(model_summary)+1] <- paste0("Adj_R2: ", adj_R2)  model_summary[length(model_summary)+1] <- ""  # Create a table containing actual and predicted values  df_val_pred <- data.frame(validation_y, y_pred)  colnames(df_val_pred) <- gsub("validation_y", "actual_y", colnames(df_val_pred))  # Generate results in the form of a list  result <- list(model_summary = model_summary, cp_check = cp_check,  df_val_pred = df_val_pred, model = m, validation_x = validation_x, validation_y = validation_y)  #################### "Model summary and validation metrics after pruning" ########################  cp <- 0.01  library(rpart)  # Data preprocessing for variables to be used in pruning  model <- result$model  cp_check <- result$cp_check  validation_x <- result$validation_x  validation_y <- result$validation_y  # Results after performing pruning  cmd <- paste0("m <- prune(model, cp =", cp, ")")  eval(parse(text = cmd))  # Generate predictions based on the created model  y_pred <- predict(m, validation_x)  # Predicted values to be used for calculating R-squared  r2_pred <- predict(m, train[r2_xvar])  # Calculate model evaluation metrics  mse <- round(mean((validation_y-y_pred)^2), 3)  rmse <- round(sqrt(mse), 3)  mae <- round(mean(abs(validation_y-y_pred)), 3)  mape <- round(mean(abs((validation_y - y_pred)/validation_y))*100, 3)  R2 <- 1-(sum((train[[yvar]]-r2_pred)^2)/sum((train[[yvar]]-mean(train[[yvar]]))^2))  R2 <- round(R2, 3)  n <- nrow(train)  k <- length(r2_xvar)  adj_R2 <- 1-((1-R2)*(n-1)/(n-k-1))  adj_R2 <- round(adj_R2, 3)  # model summary information  model_summary <- capture.output(m)  model_summary[length(model_summary)+1] <- ""  model_summary[length(model_summary)+1] <- paste0("MSE: ", mse)  model_summary[length(model_summary)+1] <- paste0("RMSE: ", rmse)  model_summary[length(model_summary)+1] <- paste0("MAE: ", mae)  model_summary[length(model_summary)+1] <- paste0("MAPE(%): ", mape, "%")  model_summary[length(model_summary)+1] <- paste0("R2: ", R2)  model_summary[length(model_summary)+1] <- paste0("Adj_R2: ", adj_R2)  model_summary[length(model_summary)+1] <- ""  #Create a table containing actual and predicted values  df_val_pred <- data.frame(validation_y, y_pred)  colnames(df_val_pred) <- gsub("validation_y", "actual_y", colnames(df_val_pred))  # Generate results in the form of a list  result <- list(model_summary = model_summary, cp_check = cp_check, df_val_pred = df_val_pred, model = m)  #################### "decision tree graph" ########################  library(ggplot2)  library(rattle)  library(rpart.plot)  library(RColorBrewer)  # Use only the model information from the results generated in the form of a list  model <- result$model  # Plot the decision tree graph  rattle::fancyRpartPlot(model, sub = "")  #################### "Result graph" ########################  library(ggplot2)  # Use only the model information from the results generated in the form of a list  model <- result$model  variable.importance <- result$model$variable.importance  variable.importance <- as.data.frame(variable.importance)  variable <- row.names(variable.importance)  df <- data.frame(variable = variable, importance = round(variable.importance[,1], 2))  # Feature importance graph  ggplot(df, aes(x=reorder(variable, importance), y= importance)) +  geom_bar(stat = "identity", fill = "steelblue") +  geom_text(aes(label = importance), hjust = -0.3, vjust = 0.3, size = 4)+  theme_minimal() +  coord_flip() +  xlab("variable")  library(ggplot2)  #Use only the actual values and predicted values from the results generated in the form of a list  actual_y <- result$df_val_pred$actual_y  y_pred <- result$df_val_pred$y_pred  # Create a table for generating a line graph (actual values)  actual_y_tmp <- data.frame(Value = actual_y,  Class = rep("actual_y", length(actual_y)),  Index = 1:length(actual_y))  # Create a table for generating a line graph (predicted values)  y_pred_tmp <- data.frame(Value = y_pred,  Class = rep("y_pred", length(y_pred)),  Index = 1:length(y_pred))  # Merge the two tables  df <- rbind(actual_y_tmp, y_pred_tmp)  # Plot a line graph for actual and predicted values  ggplot(data=df, aes(x=Index, y=Value)) + geom_line(aes(color=Class), size=1)  ############################### clsification model ####################################  #################### "Model Summary, Validation Metrics, CP Table" ########################  df <- read.csv("C:/Users/user/Downloads/filename.csv")  xvar <- colnames(df)[c()]  yvar <- colnames(df)[c()]  minbucket = 7  maxdepth = 2  train_ratio <- 70  validation_ratio <- 30  seedNum <- 1234  library(rpart)  library(caret)  library(pROC)  # Splitting the Training Data and Validation Data  set.seed(seedNum)  ratio_sum <- sum(train_ratio, validation_ratio)  train_ratio <- train_ratio / ratio_sum  # Perform stratified sampling for each class of the dependent variable  suppressWarnings(train_idx <- createDataPartition(df[[yvar]], p = train_ratio, list = F))  train <- df[train_idx, ] # Training Data  validation <- df[-train_idx, ] # Validation Data  if(train_ratio == 1) {  # Data used for training the model with training data only  validation_x <- df[xvar]  validation_y <- df[[yvar]]  } else {  # Data used for training the model with training and validation data  validation_x <- validation[xvar]  validation_y <- validation[[yvar]]  }  # Specify minbucket, maxdepth  control <- rpart.control(minbucket = minbucket, maxdepth = maxdepth)  # Create a decision tree classification model  xvar <- paste0(xvar, collapse = "+")  cmd <- paste0("m <- rpart(", yvar, " ~ ", xvar, ", data = train, control = ", control,")")  eval(parse(text=cmd))  # Check the appropriate number of terminal nodes for pruning  cp_check <- m$cptable  cp_check <- round(as.data.frame(cp_check), 8)  colnames(cp_check) <- c("Complexity(CP)", "Number of splits", "Error rate", "Cross-validation error", "Standard deviation of cross-validation error")  # Generate predictions based on the created model  y_pred <- predict(m, validation_x, type = 'class')  # Generate confusion matrix information  cm <- confusionMatrix(y_pred, validation_y)  # model summary information  model_summary <- capture.output(m, cm$table, cm$overall, cm$byClass)  # Change the class to resolve the issue of missing values where TRUE/FALSE is required  y_pred <- as.character(y_pred)  validation_y <- as.character(validation_y)  # Create a table containing actual values, predicted values, and prediction probabilities  df_val_pred <- data.frame(validation_y, y_pred)  colnames(df_val_pred) <- gsub("validation_y", "actual_y", colnames(df_val_pred))  # Generate results in the form of a list  result <- list(model_summary = model_summary, cp_check = cp_check,  df_val_pred = df_val_pred, model = m, validation_x = validation_x, validation_y = validation_y)  #################### "Model summary and validation metrics after pruning" ########################  cp <- 0.01  library(caret)  library(rpart)  # Data preprocessing for variables to be used in pruning  model <- result$model  cp_check <- result$cp_check  validation_x <- result$validation_x  validation_y <- result$validation_y  validation_y <- as.factor(validation_y)  # Results after performing pruning  cmd <- paste0("m <- prune(model, cp =", cp, ")")  eval(parse(text = cmd))  # Generate predictions based on the created model  y_pred <- predict(m, validation_x, type = 'class')  # Generate confusion matrix information  cm <- confusionMatrix(y_pred, validation_y)  # model summary information  model_summary <- capture.output(m, cm$table, cm$overall, cm$byClass)  # Change the class to resolve the issue of missing values where TRUE/FALSE is required  y_pred <- as.character(y_pred)  validation_y <- as.character(validation_y)  # Create a table containing actual and predicted values  df_val_pred <- data.frame(validation_y, y_pred)  colnames(df_val_pred) <- gsub("validation_y", "actual_y", colnames(df_val_pred))  # Generate results in the form of a list  result <- list(model_summary = model_summary, cp_check = cp_check, df_val_pred = df_val_pred, model = m)  #################### "decision tree graph" ########################  library(rattle)  library(rpart.plot)  library(RColorBrewer)  # Use only the model information from the results generated in the form of a list  model <- result$model  # Plot the decision tree graph  rattle::fancyRpartPlot(model, sub = "")  #################### "Result graph" ########################  library(ggplot2)  # Use only the model information from the results generated in the form of a list  model <- result$model  variable.importance <- result$model$variable.importance  variable.importance <- as.data.frame(variable.importance)  variable <- row.names(variable.importance)  df <- data.frame(variable = variable, importance = round(variable.importance[,1], 2))  # Feature importance graph  ggplot(df, aes(x=reorder(variable, importance), y= importance)) +  geom_bar(stat = "identity", fill = "steelblue") +  geom_text(aes(label = importance), hjust = -0.3, vjust = 0.3, size = 4)+  theme_minimal() +  coord_flip() +  xlab("variable")  library(ggplot2)  # Use only the actual values and predicted values from the results generated in the form of a list  actual_y <- result$df_val_pred$actual_y  y_pred <- result$df_val_pred$y_pred  # Generate confusion matrix  confusionM <- xtabs(~ y_pred + actual_y)  # Preprocessing for plotting the confusion matrix graph  input.matrix <- data.matrix(confusionM)  confusion <- as.data.frame(as.table(input.matrix))  colnames(confusion)[3] <- "Frequency"  # Plot the confusion matrix graph  ggplot(confusion, aes(x = actual_y, y = y_pred, fill = Frequency)) + geom_tile() +  geom_text(aes(label=confusion$Frequency)) +  scale_x_discrete(name="Actual Class") + scale_y_discrete(name="Predicted Class") +  geom_text(aes(label = Frequency),colour = "white") +  scale_fill_continuous(high = "#132B43", low = "#56B1F7") |
| --- |
